# Connectedness to Nature: Its Impact on Sustainable Behaviors and Happiness in Children

**DOI:** 10.3389/fpsyg.2020.00276

**Published:** 2020-02-26

**Authors:** Laura Fernanda Barrera-Hernández, Mirsha Alicia Sotelo-Castillo, Sonia Beatriz Echeverría-Castro, César Octavio Tapia-Fonllem

**Affiliations:** ^1^Department of Psychology, Instituto Tecnológico de Sonora, Ciudad Obregón, Mexico; ^2^Department of Psychology and Communication Sciences, Universidad de Sonora, Hermosillo, Mexico

**Keywords:** connectedness to nature, sustainable behavior, children, happiness, nature

## Abstract

Given the environmental problems humanity is currently facing, and considering that the future of the planet lies in the hands of children and their actions, research on the determinants of sustainable behaviors in children has become more relevant; nonetheless, studies on this topic focusing on children are scarce. Previous research on adults suggests, in an isolated manner, the relationship between connectedness to nature, the development of behaviors in favor of the environment, and positive results derived from them, such as happiness and well-being. In the present research, connectedness to nature was considered as a determinant of sustainable behaviors, and happiness was considered as a positive consequence of the latter. This research aimed to demonstrate the relationship between these variables in children. Two hundred and ninety-six children with an average age of 10.42 years old participated in the study, in which they responded to a research instrument that measured connectedness to nature, sustainable behaviors (*pro-ecological behavior, frugality, altruism*, and *equity*), and happiness. To analyze the relationships between these variables, a model of structural equations was specified and tested. The results revealed a significant relationship between connectedness to nature and sustainable behaviors, which, in turn, impact happiness. This suggests that children who perceive themselves as more connected to nature tend to perform more sustainable behaviors; also, the more pro-ecological, frugal, altruistic, and equitable the children are, the greater their perceived happiness will be. The implications for studying and promoting sustainable behaviors are discussed within the framework of positive psychology.

## Introduction

Environmental problems represent one of the most significant challenges humanity is currently facing, and, in such a scenario, children are important agents who could mitigate some of these environmental challenges; in their actions and in the relationship they have with the natural environment lays the opportunity to solve those problems. Hence the relevance of carrying out studies focused on this population.

This article is framed within *environmental psychology* (EP), which aims to study the relationships between behavior and the environment (Aragonés and Amérigo, 2010). Corral (2011) proposes that there are two aspects within EP: architectural psychology, which is interested in investigating the effects of the natural or built environment on behavior and its dispositions, and environmental conservation psychology, which is dedicated to studying the effects of human behavior on the environment. Olivos and Clayton (2017) point out that, although studies regarding the effects of the built environment and the *self* have a long tradition in EP, research focused on the role of nature in people’s construction of *self* and well-being were barely developed during the two first decades of the 21st century.

As environmental problems become worse, researchers are starting to focus their attention on the relationships between humans and nature and their effects on environmentally *sustainable behavior* (SB; Nisbet and Zelenski, 2013). Nisbet et al. (2009) draw attention to the fact that disconnection from the natural world may contribute to the destruction of the planet. Recently, the term *nature-deficit disorder* has been used to describe the lack of connectedness that children feel about the natural world; this concept is used to evoke a lack of a bond with other living beings (Howard, 2013). Authors like Zylstra et al. (2014) state that this disconnection from nature is mainly a consciousness problem, one that is fundamental among and for the convergent socio-ecological crises. This is of main importance for researchers and professionals concerned with environmental changes and degradation, since they see the solution to that problem as a way to develop environmental care and concern (Bruni et al., 2017).

Specialized literature offers different definitions for connectedness to nature. Mayer and Frantz (2004) conceptualize it as a trait of individuals that enables them to feel emotionally connected to the natural world. Nisbet et al. (2009) suggest the term *nature relatedness*, which they define as a relatively stable characteristic throughout time and all situations. This encompasses the appreciation and understanding of the interconnection between human beings and other living organisms and is more than love for nature or the enjoyment of its superficial facets: it includes an understanding of the importance of all of nature’s aspects, even those that are not esthetically attractive. Zylstra et al. (2014) define connectedness to nature as a stable state that includes cognitive, affective, and experiential symbiotic traits that reflect, through consistent attitudes and behaviors, sustained awareness of the interrelation between oneself and the rest of nature, which is in a continuum that includes both information and experience.

There are antecedents in the literature that have identified several benefits of connectedness to nature, including well-being (Mayer and Frantz, 2004; Nisbet and Zelenski, 2013; Zylstra et al., 2014; Olivos and Clayton, 2017), health (Nisbet and Zelenski, 2013), happiness (Nisbet and Zelenski, 2013; Zylstra et al., 2014), and a satisfying and meaningful life (Zylstra et al., 2014). Furthermore, Olivos and Clayton (2017) remark that connections with the natural environment can also have an indirect effect on well-being through pro-environmental behavior; on this matter, several studies expound that behaviors with a low environmental impact are related to happiness or satisfaction.

Several investigations reveal significant relationships between connectedness to nature and pro-ecological behaviors among the adult population (Olivos et al., 2013; Geng et al., 2015; Bruni et al., 2017). On the other hand, some researchers suggest that connectedness to nature is also related to pro-social behaviors. García et al. (2016) explain that people with a strong sense of connectedness to nature carry out a greater number of pro-ecological, frugal, fair, and altruistic behaviors, which altogether compose SB. Corral (2011) define SB as a series of actions aimed at the preservation of natural resources considering the integrity of plant and animal species, as well as the individual and social well-being of present and future generations.

Likewise, there are investigations that report a link between SB and happiness, finding a significant association between the two psychological factors and concluding that the more pro-ecological, frugal, altruistic, and equitable a person is, the more he or she experiences happiness (Corral-Verdugo et al., 2011; Tapia-Fonllem et al., 2013).

The amount of research and literature related to children’s pro-ecological attitudes and behaviors has no comparison to the number of works on the adult population, with most of the studies on this subject centered on adults (García et al., 2017). Fraijo et al. (2012) assert that it is important to arouse research interest in early age environmental education, since they consider it necessary to start aiming research efforts also at this population. Moreover, some authors point out that there are few assessments of children’s levels of connectedness to nature (Bragg et al., 2013). García et al. (2017) emphasize that studies on children could have greater effects on reducing environmental problems and increasing pro-ecological behaviors because early awareness can have better and long-lasting results.

One of the studies carried out on this topic on children is that of García et al. (2017), which expounds a relationship between connectedness to nature and pro-ecological behaviors; likewise, Collado et al. (2013) report a significant association between emotional affinity toward nature and ecological behaviors; finally, Capaldi et al. (2019) indicate that when children are in greater contact with nature, they report a greater willingness to protect it and be more pro-social.

Although previous studies have explored the relationship between connectedness to nature and pro-ecological behavior (Nisbet and Zelenski, 2013; Geng et al., 2015; Bruni et al., 2017), no studies have been conducted to address the association with the determinants of sustainable behavior except for the one performed on adults and conducted by García et al. (2016). Furthermore, despite the presence of research that has analyzed the association between sustainable behavior and happiness (Corral-Verdugo et al., 2011; Tapia-Fonllem et al., 2013), no studies were found that have proven the direct and indirect relationships between children’s connectedness to nature and their sustainable behavior and the impact of the latter on their perceived happiness.

Therefore, the present research aimed to demonstrate the relationship between connectedness to nature and sustainable behaviors, as well as the impact of these two factors on the perceived happiness of children.

## Materials and Methods

### Participants

Two hundred and ninety-six children from a northwestern Mexican city participated in this study: 175 girls and 121 boys whose ages ranged from 9 to 12 years (*M* = 10.42 years, *DE* = 1.00); 35.8% were 11 years old, 26.0% were 10 years old, 23.3% were 9 years old, and 14.9% were 12 years old. The children were enrolled in different education grades: fourth (38.5%), fifth (21.6%), and sixth (39.9%).

### Instruments

Data collection was carried out through the application of three instruments: the first was a scale to measure *connectedness to nature* (Cheng and Monroe, 2012), which consists of 16 items in the Likert scale referring to the pleasure of seeing wildflowers and wild animals, hearing sounds of nature, touching animals and plants, and considering that human beings are part of the natural world, among other rates; the scale contains five response options (from 1 = strongly disagree to 5 = totally agree).

Measurement of *sustainable behaviors* (*altruism, equity, frugality*, and *pro-ecological behavior*) was performed by adapting the scales of Fraijo et al. (2012). To assess *altruism* actions, the scale consisted of nine items that describe selfless help behaviors to other people, such as giving away used clothing, giving money to the Red Cross, helping those who fall or are hurt, among others, with five options for answering (from 1 = never to 5 = always). *Equity* was measured through seven statements that pose equality between sexes, ages, socioeconomic conditions, and races, among others; in it, participants determine their degree of agreement using a response scale (from 1 = strongly disagree to 5 = strongly agree). *Frugality* was measured by using five negative items on a Likert-type scale, which state behaviors such as using money to buy sweets, buying more food than I am going to eat, buying shoes to combine with all clothes, and so on; there were five response options (from 1 = never to 5 = always). *Pro-ecological behavior* was measured by using 11 items on the Likert scale, where participants reported the frequency (from 1 = never to 5 = always) of behaviors bound to care for the natural environment (recycling, object reuse, saving water, and separating garbage).

*Happiness* was measured by three items of the Subjective Happiness Scale (SHS; Lyubomirsky and Lepper, 1999), which measures the perception of happiness experienced by means of statements that refer to considering oneself happy in general, compared to most peers, and enjoying life regardless of what happens, with a range of answers from 1 = not very happy to 7 = very happy.

### Procedure

The instrument was self-administrated in the participants’ classroom with prior approval of the principals, teachers, and parents. Collaboration was requested from the children, explaining the purpose of the investigation to them and indicating that their participation was voluntary. The administration of the scales took about 20 min.

### Data Analysis

The results were analyzed using univariate statistics (mean, standard deviation, maximum, and minimum values); for each scale, one indicator of internal consistency was computed (Cronbach’s alpha). A matrix of correlation between the analyzed variables (connectedness to nature, altruism, equity, frugality, pro-ecological behavior, and happiness) was also obtained.

To analyze the direct and indirect relationships between these variables, a structural equation model (SEM) utilizing the EQS statistical package was specified (Bentler, 2006). The authors assumed a previous understanding of the nature and dimensionality of the items for all scales, and due to the fact that parcels can be used to optimize the measurement structure (see Little et al., 2002), the scales were parceled into three indicators for each tested construct. In order to form the parcels, the authors randomly distributed the total number of items corresponding to each factor into the indicators. The exception was happiness, wherein all three items were considered without parceling. For this study, six first-order factors were pre-specified: (1) connectedness to nature, (2) happiness, (3) altruism, (4) equity, (5) frugality, and (6) pro-ecological behavior; where the last four formed a second-order factor called “sustainable behavior.” The specified model hypothesized that the connectedness to nature factor would affect the second-order factor “sustainable behavior,” while this would positively influence happiness.

## Results

Table 1 shows the univariate statistics of the scales, as well as their internal consistency. The mean values obtained were 4.32 in connectedness to nature, 4.25 in equity, 3.54 in pro-ecological behaviors, 3.40 in altruism, and 3.15 in frugality; scales ranged from 1 to 5, representing moderate levels in pro-ecological, altruistic and frugal behaviors, and higher scores in connectedness to nature and equity. In addition, the happiness scale mean was 5.8; in this scale, the scores range from 1 to 7, which indicates that children report a high level of happiness. The Cronbach’s alpha values of the scales varied from 0.68 to 0.81, indicating an adequate level of internal consistency. The items with the highest means in *connectedness to nature* were: picking up trash from the ground can help the environment, taking care of animals is important, and human beings are part of the natural world. Regarding *altruistic behavior*, the most self-reported were: I help someone who falls or gets hurt, and I give away used clothes to the poor. On *equity*, children reported a greater degree of agreement with the statements: men and women have the same right to make decisions, boys and girls have the same opportunity to study as far as they want, and I treat poor and rich people in the same way. The most common *frugal behaviors* were: not buying more food than I am going to eat and not spending money on games and toys – on this scale, it is necessary to keep in mind that, although children have power in making family consumption decisions, parents are directly responsible for these behaviors. The most frequent *pro-ecological behaviors* were: shutting off the water faucet while brushing teeth and turning off the lights in rooms where they are not being used. Regarding the values of the responses of the happiness items, they did not differ from each other.

**TABLE 1:** Univariate statistics and reliabilities of the used scales.

	***Min***	***Max***	***Mean***	***Sd***	***Alpha***
*Connectedness to nature*	*2.25*	*5.00*	*4.32*	*0.520*	*0.81*
1. I like to hear different sounds of nature.	1	5	4.22	1.08	
2. I like to see wildflowers in nature.	1	5	4.40	0.90	
3. When I feel sad, I like to go outside and enjoy nature.	1	5	4.14	1.04	
4. Being in the natural environment makes me feel peaceful.	1	5	4.37	0.89	
5. I like to garden.	1	5	4.45	0.86	
6. Collecting rocks and shells is fun.	1	5	3.85	1.20	
7. Being outdoors makes me happy.	1	5	4.41	0.88	
8. I feel sad when wild animals are hurt.	1	5	4.43	1.02	
9. I like to see wild animals living in a clean environment.	1	5	4.58	0.81	
10. I enjoy touching animals and plants.	1	5	4.53	0.82	
11. Taking care of animals is important to me.	2	5	4.70	0.62	
12. Humans are part of the natural world.	1	5	4.59	0.80	
13. People cannot live without plants or animals.	1	5	4.55	0.93	
14. My actions will make the natural world different.	1	5	4.09	1.12	
15. Picking up trash from the ground can help the environment.	1	5	4.73	0.70	
16. People do not have the right to change the natural environment.	1	5	3.61	1.68	
*Sustainable behavior*	*2.10*	*5.00*	*3.60*	*0.511*	
*Altruism*	*1.00*	*5.00*	*3.40*	*0.827*	*0.80*
1. I give away clothes that I no longer use.	1	5	4.00	1.31	
2. I help someone who falls or gets hurt.	1	5	4.18	1.06	
3. I give money to the Red Cross.	1	5	3.64	1.24	
4. I visit sick people at the hospital.	1	5	2.50	1.40	
5. I help older or handicapped persons to cross the street.	1	5	3.02	1.44	
6. I help people to find an address.	1	5	3.38	1.37	
7. I give money to homeless people (street-living poor people).	1	5	3.49	1.37	
8. I participate in events to obtain money (fundraise) for civil organizations (firefighters, Red Cross).	1	5	2.71	1.47	
9. I explain or help schoolmates with their homework or tasks they do not understand.	1	5	3.86	1.17	
*Equity*	*2.00*	*5.00*	*4.25*	*0.641*	*0.72*
1. Men and women have the same right to make decisions about anything.	1	5	4.66	0.80	
2. I treat all my classmates as my equals.	1	5	3.99	1.11	
3. In my house, children have the same right as adults to make important decisions for the family.	1	5	3.46	1.30	
4. In my family, men and women have the same obligations in house cleaning.	1	5	4.28	1.13	
5. I treat native people in the same way as people who are not.	1	5	4.34	1.07	
6. I treat poor and rich people in the same way.	1	5	4.47	0.91	
7. In my family, girls have the same opportunity to study as boys (as far as they want).	1	5	4.64	0.86	
*Frugality*	*1.25*	*4.75*	*3.15*	*0.693*	*0.72*
1. I use my money to buy candy.	1	5	3.07	1.23	
2. I buy enough shoes and tennis to match my clothes.	1	5	2.59	1.38	
3. I buy more food than I am going to eat.	1	5	3.60	1.41	
4. At home, a lot of food is bought.	1	5	2.75	1.24	
5. I spend my money on games and toys.	1	5	3.45	1.41	
*Pro-Ecological Behavior*	*1.27*	*5.00*	*3.54*	*0.720*	*0.78*
1. I save and recycle used paper.	1	5	3.01	1.26	
2. I separate empty bottles to recycle.	1	5	3.01	1.25	
3. I tell people when their actions damage the environment.	1	5	3.74	1.23	
4. I read about nature.	1	5	3.08	1.27	
5. I look for a way to reuse things.	1	5	3.55	1.27	
6. I encourage my friends and family to recycle.	1	5	3.07	1.35	
7. When I go to nearby places, I walk or cycle.	1	5	3.77	1.34	
8. I turn off the lights in rooms where they are not being used.	1	5	4.44	1.00	
9. I shut off the water faucet while brushing my teeth.	1	5	4.53	0.99	
10. I leave the fridge door open for a long time while choosing food.	1	5	3.87	1.38	
11. I watch environmental videos or programs.	1	5	3.10	1.31	
*Happiness*	*1.00*	*7.00*	*5.81*	*1.22*	*0.68*
1. In general, I consider myself:	1	7	6.02	1.40	
2. Compared to most of the people around me, I consider myself:	1	7	5.84	1.59	
3. Some people tend to be very happy. They enjoy life in spite of what happens, facing most things. To what extent do you consider yourself such a person?	1	7	5.82	1.49	

*italics values are indicates minimum, maximum, mean, standard deviation, Cronbach Alpha.*

Table 2 exhibits the correlations between connectedness to nature, happiness, and the determinants of sustainable behavior (altruism, equity, frugality, and pro-ecological behavior). The highest correlation with happiness occurred between this factor and connectedness to nature, followed by those with altruism, pro-ecological behavior, equity, and frugality. The lowest association occurred between frugality and happiness.

**TABLE 2:** Correlations between variables.

	**CNAT**	**ALT**	**EQU**	**FRU**	**PEB**	**SB**	**HAP**
Connectedness to nature	1						
Altruism	0.43**	1					
Equity	0.39**	0.26**	1				
Frugality	0.18**	0.09	0.15**	1			
Pro-ecological behavior	0.49**	0.61**	0.34**	0.26**	1		
Sustainable behavior	0.54**	0.76**	0.61**	0.53**	0.82**	1	
Happiness	0.31**	0.25**	0.10	–0.05	0.19**	0.19**	1

**p < 0.001. CNAT, connectedness to nature; ALT, altruism; EQU, Equity; FRU. Frugality; PEB, pro-ecological behavior; SB, sustainable behavior, HAP, happiness.

**FIGURE 1:**
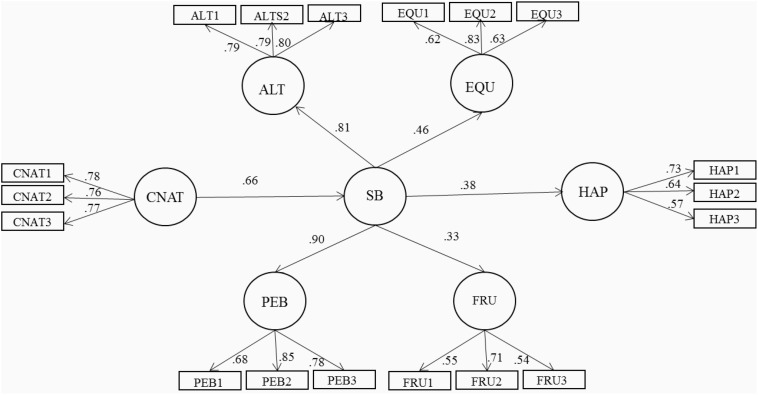
Structural model of sustainable behavior and happiness, predicted by connectedness to nature. Goodness of fit: *X*^2^ = 203.26 (125 *d.f.*) *p* = 0.000, *BBNFI* = 0.90, *BBNNFI* = 0.94, *CFI* = 0.95; *RMSEA* = 0.04; R^2^ Sustainable behavior = 0.42; R^2^ Happiness = 0.14.

Figure 1 exhibits the results of the structural model evaluating the relationship between connectedness to nature, sustainable behavior, and happiness. The factor loadings that connected the first-order factors with their corresponding indicators were high and significant (*p* < 0.05), revealing convergent construct validity for the used measures. Furthermore, the first-order factors (pro-ecological behavior, altruism, frugality, and equity) correlated significantly with their corresponding second-order factor (sustainable behavior), as revealed by the value and statistical significance (*p* < 0.05) of their factorial loadings. In the model of structural equations, it was found that connectedness to nature (structural coefficient = 0.66; *p* < 0.05) influences sustainable behavior, and in turn, as expected, this positively affects happiness (structural coefficient = 0.38; *p* < 0.05). Having said that, although the chi-square value (*X*^2^ = 203.26, 125 *df*) associated to this model was significant (*p* < 0.0001), the values of the practical indices Bentler Bonett Normed Fit Index (BBNFI; = 0.90), Bentler Bonnet Non-normed Fit Index (BBNNFI; = 0.94), Comparative Fit Index (CFI; = 0.95), as well as RMSEA (0.04), support the pertinence of this interrelations model.

## Discussion

The present study is framed within the positive sustainability psychology line of research, which studies the antecedents and positive consequences of pro-environmental or sustainable behaviors (Corral-Verdugo et al., 2011); according to Adams and Savahl (2017), there is a tendency to merge the theories of EP, sustainability, and positive psychology, emphasizing the importance that the natural environment commitment has in people’s well-being.

As previous research and our results show, there is a relationship between feeling connected to nature and carrying out sustainable behaviors (García et al., 2016) and also between carrying out sustainable behaviors and the happiness of individuals (Corral-Verdugo et al., 2011), and this also applies to children. In addition to the above, the most remarkable findings of the study reported here include the associations between connectedness to nature, the four determinants of sustainable behavior (altruism, equity, frugality, and pro-ecological behavior), and happiness. A possible exception to this conclusion is the relationship between frugality and happiness. Although frugal behaviors were correlated to the rest of the sustainable behavior indicators, and this aggregate of actions significantly predicted happiness, the matrix correlation revealed that frugality and happiness presented an almost-zero correlation. These findings agree with the results reported by previous research (Corral-Verdugo et al., 2011). One explanation for the lack of relationship between frugality and happiness could be that frugality may not have been voluntary, because although children influence consumption decisions, parents make the ultimate choice in these matters. Future studies may investigate this relationship by contrasting the correlations between happiness and voluntary frugality across age samples.

The findings of the model tested revealed that connectedness to nature impacts sustainable behaviors and that these result in happiness. This suggests that children who perceive themselves as more connected to nature tend to perform more sustainable behaviors, and the more pro-ecological, altruistic, frugal, and equitable the child is, the greater his or her perceived happiness will be. These results confirm findings presented in previous research carried out with adults, in the sense that connectedness to nature leads to performing protective behaviors for the sake of the physical (Nisbet and Zelenski, 2013; Geng et al., 2015; Bruni et al., 2017) and social environments (García et al., 2016, 2017), which in turn generate happiness (Corral-Verdugo et al., 2011; Tapia-Fonllem et al., 2013). However, none of those studies were focused on investigating these variables and the relationship between them in children; so, part of the purpose of this study was to contribute to fixing that gap in the specialized literature.

It is necessary to mention several limitations of this study: the number of participants, their age, and the fact that they lived in the same city; altogether, this makes it impossible to conclude that the sample is representative of the Mexican population aged 9 to 12 years. Besides, in spite of differences that may exist between socio-demographic data such as sex, age, and school grade, the results were not compared based on those characteristics. Furthermore, the use of self-report scales in the measurement of variables presents disadvantages compared to other, more objective data-collection techniques (e.g. observations, third-party reports, behavioral traces). In addition, in the measurement of frugality, although children have the power to make family consumption decisions, parents are directly responsible for these behaviors. Finally, the correlational research design could also be considered as a limitation, given its restrictions compared with experimental studies.

Despite these limitations, the findings of this study provide an advance in the knowledge about the positive psychology of sustainability in children, deepening it into the relationships between connectedness to nature, sustainable behaviors, and happiness. Fraijo et al. (2012) highlight the intention to look after future generations and not only for the present one, which should be taken into consideration in the study of sustainable behaviors. This study was focused on children, because the need to foster love for nature in them is increasingly being recognized nowadays, as children will be the future caretakers of natural places, and because it is from the love to nature that arises the need to protect it (Bragg et al., 2013).

Future research could solve the limitations of the present study, replicate the findings, and address what was proposed by Otto and Pensini (2017), who suggest further investigation of nature-based environmental education to promote ecological motivation in people, connectedness to nature, and environmental knowledge as complementary factors of ecological behavior. In addition, the development of research focused on educational and recreational interventions based on nature, including long-term follow-up (Bragg et al., 2013), should be considered, as should the documentation of tangible actions to move toward reconnection with nature, in order to collect evidence that supports and encourages these lines of research (Ives et al., 2018).

## Data Availability Statement

The datasets generated for this study are available on request to the corresponding author.

## Ethics Statement

This study was reviewed and approved by the ethical board of Instituto Tecnológico de Sonora. Written informed consent to participate in this study was provided by the participants’ legal guardian/next of kin.

## Author Contributions

CT-F and LB-H designed the project and supervised the findings of this study. MS-C and LB-H performed the data collection and analyzed the data. LB-H and SE-C wrote the manuscript with input from all other authors.

## Conflict of Interest

The authors declare that the research was conducted in the absence of any commercial or financial relationships that could be construed as a potential conflict of interest.
